# Quality of Life and Mental Health Status Among Cancer Patients With Metastatic Spinal Disease

**DOI:** 10.3389/fpubh.2022.916004

**Published:** 2022-07-05

**Authors:** Yaosheng Liu, Xuyong Cao, Xiongwei Zhao, Xiaolin Shi, Mingxing Lei, Haifeng Qin

**Affiliations:** ^1^Senior Department of Orthopedics, The Fourth Medical Center of PLA General Hospital, Beijing, China; ^2^National Clinical Research Center for Orthopedics, Sports Medicine and Rehabilitation, PLA General Hospital, Beijing, China; ^3^Department of Orthopedic Surgery, The Fifth Medical Center of PLA General Hospital, Fifth School of Clinical Medicine, Anhui Medical University, Hefei, China; ^4^Department of Orthopedic Surgery, The Fifth Medical Center of PLA General Hospital, Beijing, China; ^5^Department of Orthopedic Surgery, The Second Affiliated Hospital of Zhejiang Chinese Medical University, Hangzhou, China; ^6^Chinese PLA Medical School, Beijing, China; ^7^Department of Orthopedic Surgery, Hainan Hospital of PLA General Hospital, Sanya, China; ^8^Senior Department of Oncology, The Fifth Medical Center of PLA General Hospital, Beijing, China

**Keywords:** advanced cancer, quality of life, anxiety, depression, age

## Abstract

This study aimed to investigate the quality of life and mental health status and further to identify relevant risk factors among advanced cancer patients with spine metastases. This study prospectively included and analyzed 103 advanced cancer patients with spine metastases. Patient's basic information, lifestyles, comorbidities, tumor characteristics, therapeutic strategies, economic conditions, quality of life, anxiety, and depression were collected. Patient's quality of life was assessed using the Functional Assessment of Cancer Therapy-General Scale (FACT-G), and anxiety and depression were evaluated using the Hospital Anxiety and Depression Scale (HADS). Subgroup analysis was performed based on different age groups, and a multivariate analysis was performed to test the ability of 20 potential risk factors to predict quality of life, anxiety, and depression. The mean total FACT-G score was only 61.38 ± 21.26. Of all included patients, 52.43% had skeptical or identified anxiety and 53.40% suffered from skeptical or identified depression. Patients had an age of 60 or more and <70 years had the lowest FACT-G score (54.91 ± 19.22), highest HADS anxiety score (10.25 ± 4.22), and highest HADS depression score (10.13 ± 4.94). After adjusting all other potential risk factors, age was still significantly associated with quality of life (OR = 0.57, 95%CI: 0.38–0.86, *p* < 0.01) and depression (OR = 1.55, 95%CI: 1.00–2.42, *p* = 0.05) and almost significantly associated with anxiety (OR = 1.52, 95%CI: 0.94–2.43, *p* = 0.08). Besides, preference to eating vegetables, time since knowing cancer diagnosis, surgical treatment at primary cancer, hormone endocrine therapy, and economic burden due to cancer treatments were found to be significantly associated with the quality of life. A number of comorbidities and economic burden due to cancer treatments were significantly associated with anxiety. Advanced cancer patients with spine metastases suffer from poor quality of life and severe anxiety and depression, especially among patients with an age of 60 or more and <70 years. Early mental health care and effective measures should be conducted to advanced cancer patients with spine metastases, and more attention should be paid to take care of patients with an age of 60 or more and <70 years in terms of their quality of life and mental health status.

## Introduction

Cancer caused almost 10.0 million deaths worldwide in 2020 and the global cancer burden is estimated to be 28.4 million cases in 2040, an up to 47% increase from 2020 (1). The life expectancy of patients with cancer is continuing to rise due to the advances in early detection and treatment (2). However, patients with advanced cancer generally suffered from poor quality of life and severe mental disorders: 23.4–32.0% had anxiety and 19.1–47.0% lived with depression (3, 4). Anxiety and depression contribute to additional burdens even elevated premature mortality (5, 6), making it more challenging regarding its management and control (7).

Spine is one of the most common sites for the involvement of metastatic disease (8, 9). As an advanced stage of cancer, metastatic spinal disease is often characterized by severe back pain, infection, neurological sequelae, and ambulatory disability (8), which are particularly challenging for surgeons. Of note, patients with spine metastasis have been proved to be relevant to declined life expectancy (8) and poor quality of life (10–12) because of above complications. Studies have demonstrated that surgical interventions could be capable of improving the quality of patient's remaining life due to immediate pain relief and functional recovery among patients with spine metastasis (10–12).

Healthcare interventions can be individually conducted under the guide of predictors, thus identifying that the predictors of quality of life will facilitate the recognition of patients who benefit most from early therapeutic techniques. Several features have been found to be associated with quality of life especially among patients with metastatic spial disease after surgery, such as the Frankel grade (13), Karnofsky performance score (13), motor weakness (14), severity of pain (14), and bowel or bladder dysfunction (14). These features would be of great help to aid surgeons to make patient selection and education, and setting treatment expectations, and identification of more new modifiable predictors could further provide more accurate and useful information for surgeons and patients to make clinical therapeutic decisions.

However, studies about mental distress were scarce, especially among advanced cancer patients with spine metastasis. The majority of studies focused on the investigation of mental status in patients with general cancer (15, 16) or a specific cancer type (17–19). Studies have reported that age (18, 19), gender (15), income (18), primary cancer type (15), tumor stage (18), marital status (18), occupation status (18), social support (16), and appraisal of illness (16) were closely linked to depression, and age (15, 18, 19), gender (15), number of children (18), income (18), tumor stage (18), marital status (18), occupation status (18), primary cancer type (15), and tumor stage (18) were significantly relevant to anxiety. Nonetheless, some studies also demonstrated that age had no significant effects on affecting patient's quality of life and mental issues (16, 20). Therefore, whether and how age plays a role in influencing quality of life and mental health warrants to be further investigated. More importantly, the above-mentioned studies were not especially designed for patients with spine metastasis, and thus, the above-mentioned risk factors might not be applicable among those patients. Identification of relevant risk factors in a particular population would be greatly beneficial to health care and elucidation of the above questions will be able to guide medical workers to implement proper interventions and strategies for preventing poor quality of life and mental distress.

Therefore, this study aimed to investigate the quality of life and mental health status and further to identify the independent risk factors associating with quality of life and mental disorders especially among cancer patients with spine metastases.

## Patients and Methods

### Patients and Study Design

This prospective cross-sectional study included and analyzed 103 patients with spine metastases between April 2021 and October 2021 in the Fifth Medical Center of PLA General Hospital (Beijing, China). Patients were asked to be voluntary to participate in an online survey, and the survey was completed in a face-to-face approach. Patients completed the questionnaire in the survey according to their actual conditions, and the survey could only be submitted if patients completed the survey entirely. This study did not take any financial compensation strategies to lure patients to take part in the survey because this might lead to patient's selection bias. If patients had any problems during the survey, doctors or researchers could be always available to them. The survey collected patient's basic information, lifestyles, comorbidities, tumor characteristics, therapeutic strategies, economic conditions, quality of life, and mental health problems. Personal information was anonymous to protect the confidentiality in the questionnaire. The questionnaire contains about 20 questions and two scales (Supplementary File S1) and it takes about 15 min to complete.

Patients agreed to participate were included and only patients with spine metastasis were included in the analysis. Patients had a histologically confirmed cancer and metastatic disease was proved by radiographic image or biopsy. Patients were excluded based on the following criteria: (1) patients aged <18 years; (2) the primary cancer site was unknown; (3) patients were reluctant to take part in the survey; (4) patients had other bone metastasis except for spine; (5) patients lost consciousness or could not collaborate with doctors or researchers to complete the survey. Supplementary Figure S1 shows the patient's flowchart. This study was approved by the Ethics Committee of the Fourth Medical Center of PLA General Hospital. Informed written consent was obtained from all patients and all data were anonymously collected. All respondents were informed of the purpose of the research and were completely voluntary to participate in the research. This study complied with the Declaration of Helsinki.

### Evaluation of Quality of Life, Anxiety, and Depression

Patient's quality of life was assessed using the Functional Assessment of Cancer Therapy-General Scale (FACT-G) (21, 22). The FACT-G is a widely used scale to evaluate the quality of life among patients with various types of cancer. It includes four main subdomains, namely, physical wellbeing, social/family wellbeing, emotional wellbeing, and functional wellbeing. The physical wellbeing, social/family wellbeing, and functional wellbeing have seven items and the emotional wellbeing has six items. Participants are asked to fill each item according to a 5-point Likert scale, ranging from 0 indicating “not at all” to 4 indicating “very much.” The scale was responded by patients according to their real conditions within previous 7 days. We obtained scores based on the FACT-G scoring guidelines and the scores of reverse items were subtracted from 4. The range of total scores of the FACT-G is from 0 to 108 by adding the four subdomains with a higher score indicating a better quality of life. Chinese vision of FACT-G was used in the study to enable patients to understand the survey.

Anxiety and depression were evaluated using the Hospital Anxiety and Depression Scale (HADS) (23), which is widely used among patients with cancer (24). The HADS consists of 14 items, including 7 items for HADS anxiety and 7 items for HADS depression. Each item includes a four-stage response format and patients complete each question *via* self-reports at the time of recruitment. The both scales range from 0 to 21, with a higher score indicating a higher severity of anxiety and depression. Patients with a total score of 8–10 in each subscale are considered having skeptical anxiety or depression and patients with a total score of 11 or more are considered as having identified anxiety or depression. Chinese vision of HADS was used in the study to enable patients to understand the survey.

### Subgroup Analysis of Groups Based on Age

Patients were divided into four subgroups in terms of age: patients aged <50 years (Group A); patients aged 50 and below 60 years (Group B); patients aged 60 and <70 years (Group C); and patients aged 70 or more years (Group D). In this classification, every 10 years was regarded as an age stage. Moreover, to further visualize the data and analyze the relationship between age and outcomes in a more accurate and concise approach, patients were classified into seven different groups in terms of their age, and in this classification, every 5 years was considered as an age stage: <50 years (Group a); ≧50 and <55 years (Group b); ≧55 and <60 years (Group c); ≧60 and <65 years (Group d); ≧65 and <70 years (Group e); ≧70 and <75 years (Group f); and ≧75 years (Group g). Then, this study performed subgroup analyses of FACT-G score and its four subscales (physical wellbeing score, social/family wellbeing score, emotional wellbeing score, and functional wellbeing score). Besides, HADS anxiety and HADS depression scores were also compared between those age subgroups.

### Internal Consistency and Correlation of the Scales

Internal consistency of the FACT-G and HADS was conducted by calculating Cronbach's α coefficient and comparing with other studies (25, 26). A Cronbach's α coefficient of more than 0.7 is considered as acceptable and 0.8 or more are regarded as excellent for an instrument's internal reliability. Correlation of the scales was evaluated using the Spearman's correlation coefficients. Besides, correlation coefficients of the scales were also calculated using the multiply linear regression models adjusted for patient's age, sex, number of comorbidities, and primary cancer type (17).

### Multivariate Analysis of Potential Risk Factors

A multivariate analysis was performed to test the ability of 20 potential risk factors to predict the quality of life, anxiety, and depression. These risk factors included age (years) (<50 vs. ≧50 and <60 vs. ≧60 and <70 vs. ≧70), sex (male vs. female), education level (primary or junior school vs. high school vs. undergraduate vs. graduate or above), care giver (spouse vs. other family member vs. nursing worker vs. none), preference to eating meat (yes vs. no), preference to eating vegetables (yes vs. no), preference to eating fruits (yes vs. no), addiction to smoking (yes vs. no vs. abstain from smoking), addiction to drinking (yes vs. no vs. abstain from drinking), number of comorbidities (0 vs. 1 vs. 2 vs.≧3), time since knowing cancer diagnosis (months) (<3 vs. ≧3 and <6 vs. ≧6 and <12 vs. ≧12), primary cancer type (lung cancer vs. liver cancer vs. breast cancer vs. prostate cancer vs. digestive tract cancer vs. others), visceral metastasis (yes vs. no), surgical treatment at primary cancer (open surgery vs. minimally invasive surgery vs. none), surgical treatment at spine metastasis (open surgery vs. minimally invasive surgery vs. none), radiotherapy (yes vs. no), chemotherapy (yes vs. no), hormone endocrine therapy (yes vs. no), disposable monthly income [Yuan, Renminbi (RMB)] (<5,000 vs. ≧5,000 and <10,000 vs. ≧10,000 and <20,000 vs. ≧20,000), and economic burden due to cancer treatments (none vs. mild vs. moderate vs. severe). The above characteristics were reported by enrolled patients. Comorbidities included hypertension, diabetes, coronary heart disease, cataract, glaucoma, chronic liver disease, benign prostatic hyperplasia, chronic kidney disease, cerebral vascular disease, asthma, chronic bronchitis, and others. The time since knowing cancer diagnosis was defined as the time intervals between the date of cancer diagnosis knew to the patients and the date that the patients participated in the survey.

### Statistical Analysis

Descriptive statistics were used to describe the whole sample. Continuous characteristics were presented as mean ± standard deviation and categorical characteristics were presented as frequency and/or percentage. Analysis of variance, supplied by the Kruskal–Wallis rank test, was used to assess the difference between different age groups. Multiple comparisons within age groups were achieved using the Tukey's test. Cronbach's α coefficient and correlations of the scales were also calculated. The multivariate analyses were achieved using the multiply logistic regression models. When conducting the multiply logistic regression models, the FACT-G was classified as <40, ≧40 and <50, ≧50 and <60, ≧60 and <70, ≧70 and <80, ≧80 and <90, ≧90 and <100, and ≧100, both anxiety and depression were classified as none, skeptical, and identified anxiety or depression, and four age subgroups were used in the analysis. We further assessed the area under the receiver operating characteristic curve (AUROC) value of significant variables alone or combined. A database was extracted and constructed based on the survey, and all statistical analyses were conducted in the database. Statistical significance was set at a *p-*value of 0.05 or less with two-sided tests. All data processing, statistical analysis, and plotting were conducted in R 4.0.5 software and SAS 9.4 for Windows XP.

## Results

### Patient's Demographics

In the entire cohort of patients, the mean age was 59.22 ± 12.79 years and there were 52.43% male patients. The majority of patients had primary or junior school education background (40.78%, 42/103) and a multitude of caregivers were patient's spouse (61.17%, 63/103). Of all the enrolled patients, 58.25% (60/103) of patients did not receive any surgical treatment at primary cancer, but 62.14% (64/103) of patients were performed with minimally invasive surgery at spine metastasis. Regarding economic burden, 51.46% (53/103) patients thought that cancer treatments triggered severe economic pressure. Most of the patients (63.11%, 65/103) had already known that he or she had diagnosed with cancer for 1 year. The mean total FACT-G score was only 61.38 ± 21.26, indicating that those patients suffered from poor quality of life. Besides, the burden of anxiety and depression was also heavy. Of all included patients, 52.43% had skeptical or identified anxiety and 53.40% suffered from skeptical or identified depression. More details are shown in Table 1.

**Table 1:** Demographics and clinical characteristics among advanced cancer patients with spine metastases.

**Characteristics**	**Patients (*n* = 103)**
Age (mean, years)	59.22 ± 12.79
Sex	
Man	52.43%
Woman	47.57%
Education level	
Primary or junior school	40.78%
High school	28.16%
Undergraduate	28.16%
Graduate or above	2.91%
Caregiver	
Spouse	61.17%
Other family member	23.30%
Nursing worker	5.83%
None	9.71%
Preference to eating meat	
Yes	62.14%
No	37.86%
Preference to eating vegetables	
Yes	88.35%
No	11.65%
Preference to eating fruits	
Yes	47.57%
No	52.43%
Addiction to smoking	
Yes	16.50%
No	57.28%
Abstain from smoking	26.21%
Addiction to drinking	
Yes	6.80%
No	74.76%
Abstain from drinking	18.45%
Number of comorbidities	
0	66.99%
1	16.50%
2	9.71%
≧3	6.80%
Time since knowing cancer diagnosis (months)	
<3	15.53%
≧3 and <6	8.74%
≧6 and <12	12.62%
≧12	63.11%
Primary cancer type	
Lung cancer	57.28%
Liver cancer	4.85%
Breast cancer	4.85%
Prostate cancer	1.94%
Digestive tract cancer	9.71%
Others	21.36%
Visceral metastasis	
Yes	38.83%
No	61.17%
Surgical treatment at primary cancer	
Open surgery	15.53%
Minimally invasive surgery	26.21%
None	58.25%
Surgical treatment at spine metastasis	
Open surgery	14.56%
Minimally invasive surgery	62.14%
None	23.30%
Radiotherapy	
Yes	60.19%
No	39.81%
Chemotherapy	
Yes	56.31%
No	43.69%
Hormone endocrine therapy	
Yes	13.59%
No	86.41%
Disposable monthly income (Yuan, RMB)	
<5,000	66.02%
≧5,000 and <10,000	22.33%
≧10,000 and <20,000	4.85%
≧20,000	6.80%
Economic burden due to cancer treatments	
None	2.91%
Mild	10.68%
Moderate	34.95%
Severe	51.46%
Physical wellbeing	14.38 ± 7.22
Social/family wellbeing	19.33 ± 5.71
Emotional wellbeing	14.21 ± 5.90
Functional wellbeing	13.46 ± 7.37
Total FACT-G score	61.38 ± 21.26
HADS-anxiety	8.51 ± 4.65
None	47.57% (49/103)
Skeptical	19.42% (20/103)
Identified	33.01% (34/103)
HADS-depression	8.36 ± 5.22
None	46.60% (48/103)
Skeptical	20.39% (21/103)
Identified	33.01% (34/103)

*RMB, Renminbi; FACT-G, Functional Assessment of Cancer Therapy-General Scale; HADS, Hospital Anxiety and Depression Scale*.

### Subgroup Analysis of Groups Based on Age

**Figure 1:**
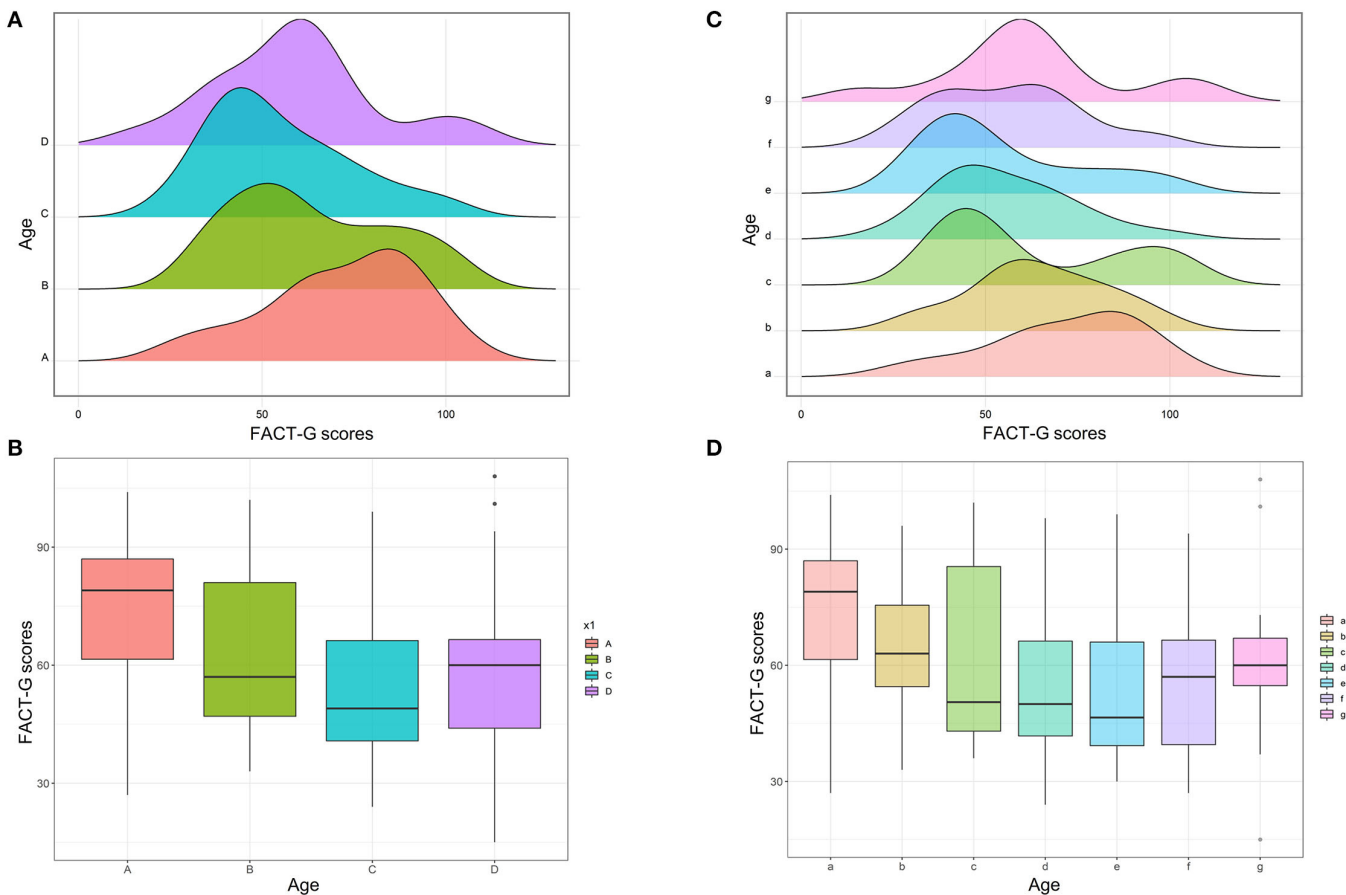
Subgroup analysis of age for FACT-G scores. **(A)** Histogram of FACT-G scores between four age subgroups (Group A, <50 years; Group B, ≧50 and <60 years; Group C, ≧60 and <70 years; Group D, ≧70 years). **(B)** Box plot of FACT-G scores between the four age subgroups. **(C)** Histogram of FACT-G scores between seven age subgroups (Group a, <50 years; Group b, ≧50 and <55 years; Group c, ≧55 and <60 years; Group d, ≧60 and <65 years; Group e, ≧65 and <70 years; Group f, ≧70 and <75 years; Group g, ≧75 years). **(D)** Box plot of FACT-G scores between the seven age subgroups.

A scatter diagram was drawn for age against the FACT-G scores, and a smooth line was fitted according to the linear regression model (FACT-G scores = −0.4047^*^Age + 85.35, Supplementary Figure S2). It demonstrated that the age and FACT-G scores were negatively correlated in general. Moreover, to find more detail information, this study performed multiple comparisons between age subgroups. First, patients were divided into four subgroups according to their age. Patients aged above or equal to 60 but <70 years (Group C) had the lowest total mean FACT-G scores, as compared to other three age subgroups (*p* = 0.04, Table 2). The data were visualized using the histogram (Figure 1A) and the box plot (Figure 1B). The peak of group C (patients aged above or equal to 60 but less than 70 years) was located at the far left, indicating that those patients had lowest FACT-G scores, which was also demonstrated by the box plot. Generally, it demonstrated that FACT-G scores were decreasing with age, reaching it bottom, and then increasing with age. A similar trend was also observed in physical wellbeing scores (*p* = 0.02) and functional wellbeing scores (*p* < 0.01) (Table 2). Although the social wellbeing scores in the patients aged above or equal to 60 but less than 70 years were the lowest, compared with that in other three age subgroups, it reached no significance (*p* = 0.16). As for both HADS anxiety and HADS depression scores, patients with an age of 60 or more and less than 70 years spiked (both, *p* < 0.01), as compared to other three age subgroups (Table 2).

**Table 2:** Differences of quality of life, anxiety, and depression among various age subgroups in patients with spine metastases.

**Scores**	**Age (years)**				**P**
	** <50**	**≧50 and <60**	**≧60 and <70**	**≧70**	
Physical wellbeing	18.42 ± 7.20	14.90 ± 7.30	12.03 ± 5.81	13.65 ± 7.94	0.02
Social/family wellbeing	19.21 ± 6.07	20.31 ± 6.23	19.41 ± 5.73	18.09 ± 4.91	0.36
Emotional wellbeing	16.84 ± 5.10	13.97 ± 5.89	13.00 ± 5.84	14.04 ± 6.44	0.16
Functional wellbeing	17.37 ± 5.61	14.48 ± 7.99	10.47 ± 7.04	13.09 ± 7.04	<0.01
Total FACT-G score	71.84 ± 20.77	63.66 ± 21.33	54.91 ± 19.22	58.87 ± 22.26	0.04
HADS-anxiety	5.47 ± 3.03	8.38 ± 5.12	10.25 ± 4.22	8.78 ± 4.77	<0.01
HADS-depression	4.79 ± 3.54	8.14 ± 5.50	10.13 ± 4.94	9.13 ± 5.29	<0.01

*FACT-G, Functional Assessment of Cancer Therapy-General Scale; HADS, Hospital Anxiety and Depression Scale*.

**Figure 2:**
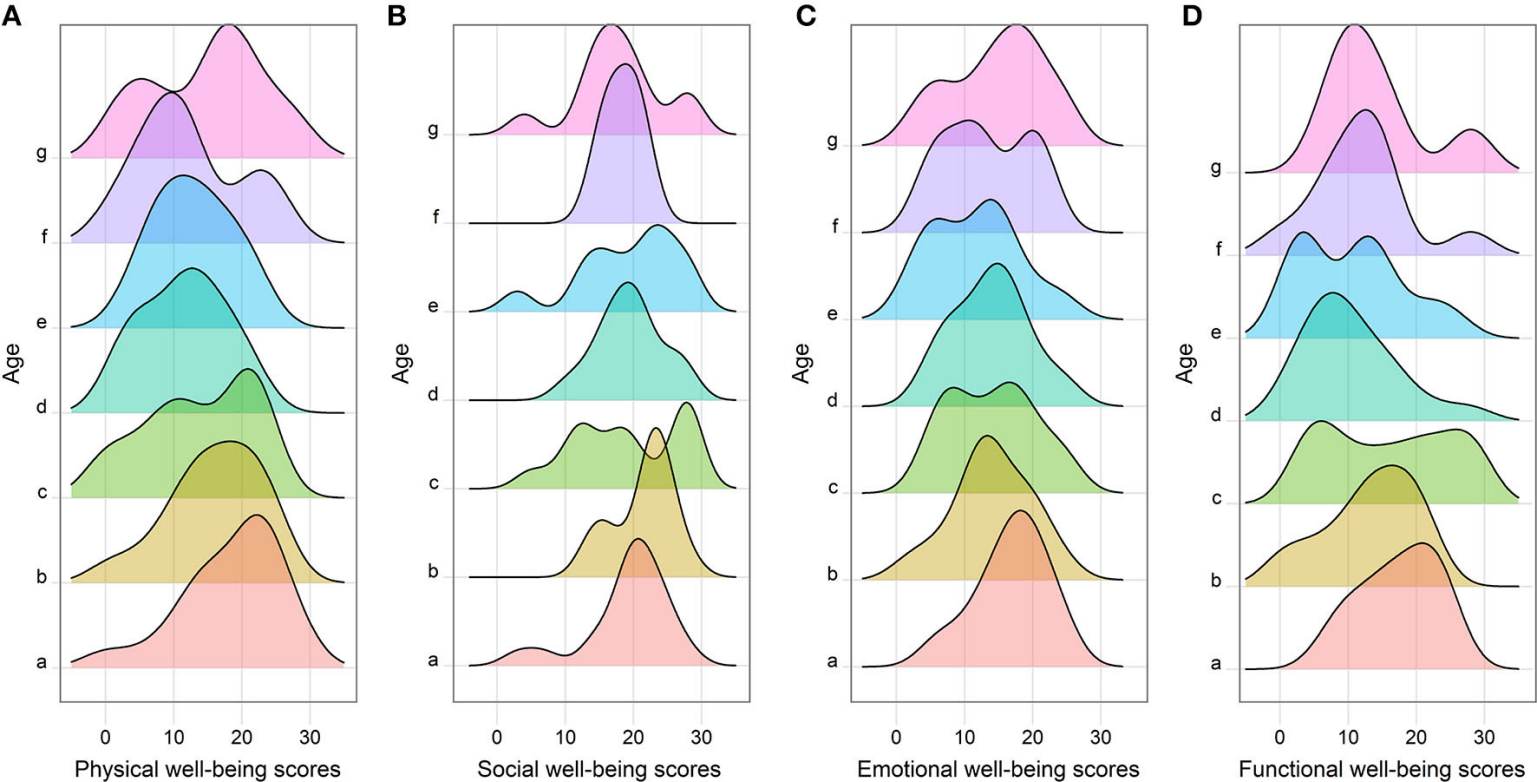
Histogram of FACT-G subscales between the seven age subgroups (Group a, <50 years; Group b, ≧50 and <55 years; Group c, ≧55 and <60 years; Group d, ≧60 and <65 years; Group e, ≧65 and <70 years; Group f, ≧70 and <75 years; Group g, ≧75 years), including physical wellbeing scores **(A)**, social wellbeing scores **(B)**, emotional wellbeing scores **(C)**, and functional wellbeing scores **(D)**.

**Figure 3:**
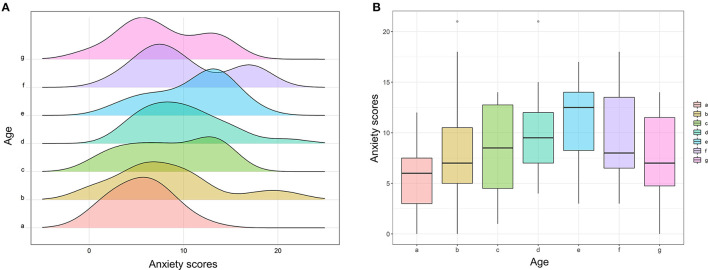
Subgroup analysis of age for anxiety scores. **(A)** Histogram of anxiety scores between seven age subgroups (Group a, <50 years; Group b, ≧50 and <55 years; Group c, ≧55 and <60 years; Group d, ≧60 and <65 years; Group e, ≧65 and <70 years; Group f, ≧70 and <75 years; Group g, ≧75 years). **(B)** Box plot of anxiety scores between the seven age subgroups.

**Figure 4:**
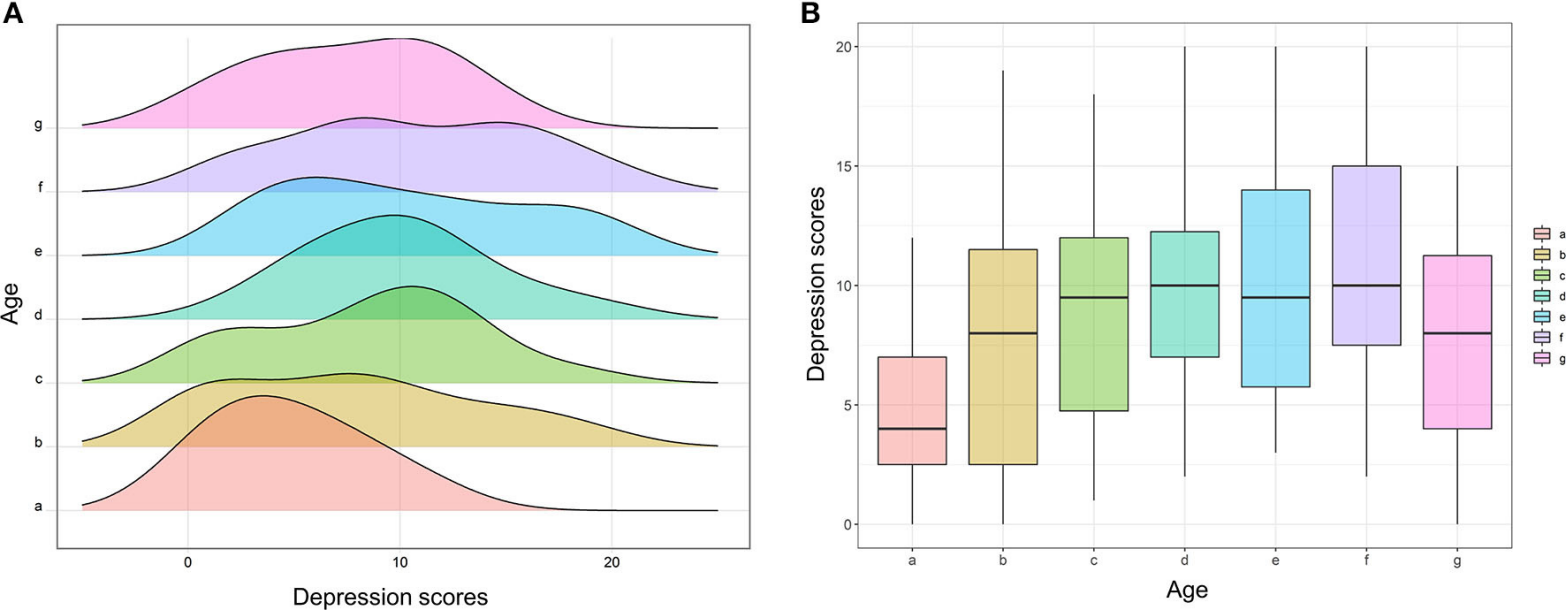
Subgroup analysis of age for depression scores. **(A)** Histogram of depression scores between seven age subgroups (Group a, <50 years; Group b, ≧50 and <55 years; Group c, ≧55 and <60 years; Group d, ≧60 and <65 years; Group e, ≧65 and <70 years; Group f, ≧70 and <75 years; Group g, ≧75 years). **(B)** Box plot of depression scores between the seven age subgroups.

Furthermore, to further investigate the relationship between age and outcomes in a more accurate and concise approach, this study divided all patients into seven subgroups according to their age: <50 years (Group a); ≧50 and <55 years (Group b); ≧55 and <60 years (Group c); ≧60 and <65 years (Group d); ≧65 and <70 years (Group e); ≧70 and <75 years (Group f); and ≧75 years (Group g). Figure 1C shows that the peak of Group e was located at the far left and gradually moved to right with increasing or decreasing age. Figure 1D also shows that the FACT-G score was declining with the increasing age, hits its bottom at Group e, and then increased with the growth of age. The similar trend was also observed in physical wellbeing scores (Figure 2A), emotional wellbeing scores (Figure 2C), and functional wellbeing scores (Figure 2D), but social wellbeing scores (Figure 2B). The opposite trend was observed in both HADS anxiety scores (Figure 3) and HADS depression scores (Figure 4): HADS scores increased with patient's age, reached its spike at Group e or f, and then decreased as age growing. The bottom of the FACT-G score and the spike of the HADS were both at patients with an age of about 65 years. The above-mentioned results indicated that patients with an age of about 65 years had a very poor quality of life and severe mental health status.

### Internal Consistency and Correlation of Scales

The FACT-G and HADS and their subscales were tested for the ability of internal consistency using Cronbach's α coefficient (Table 3). The Cronbach's α coefficient ranges from 0.82 in social/family wellbeing to 0.91 in physical wellbeing. The total FACT-G score was up to 0.94. Both HADS anxiety and HADS depression were 0.88. The results were comparable to those obtained by Sanchez et al. (25) and Bjelland et al. (26). No single item placed a significant impact in the scale alpha when it was removed.

**Table 3:** Internal consistency of the FACT-G and HADS using Cronbach's α coefficient.

**Scores**	**Current study**	Other studies^*^
Physical wellbeing	0.91	0.85
Social/family wellbeing	0.82	0.79
Emotional wellbeing	0.84	0.85
Functional wellbeing	0.89	0.73
Total FACT-G score	0.94	0.89
HADS-anxiety	0.88	0.68–0.93
HADS-depression	0.88	0.67–0.90

* *Indicates the Cronbach α coefficient obtained from previous studies. FACT-G, Functional Assessment of Cancer Therapy-General Scale; HADS, Hospital Anxiety and Depression Scale*.

**Figure 5:**
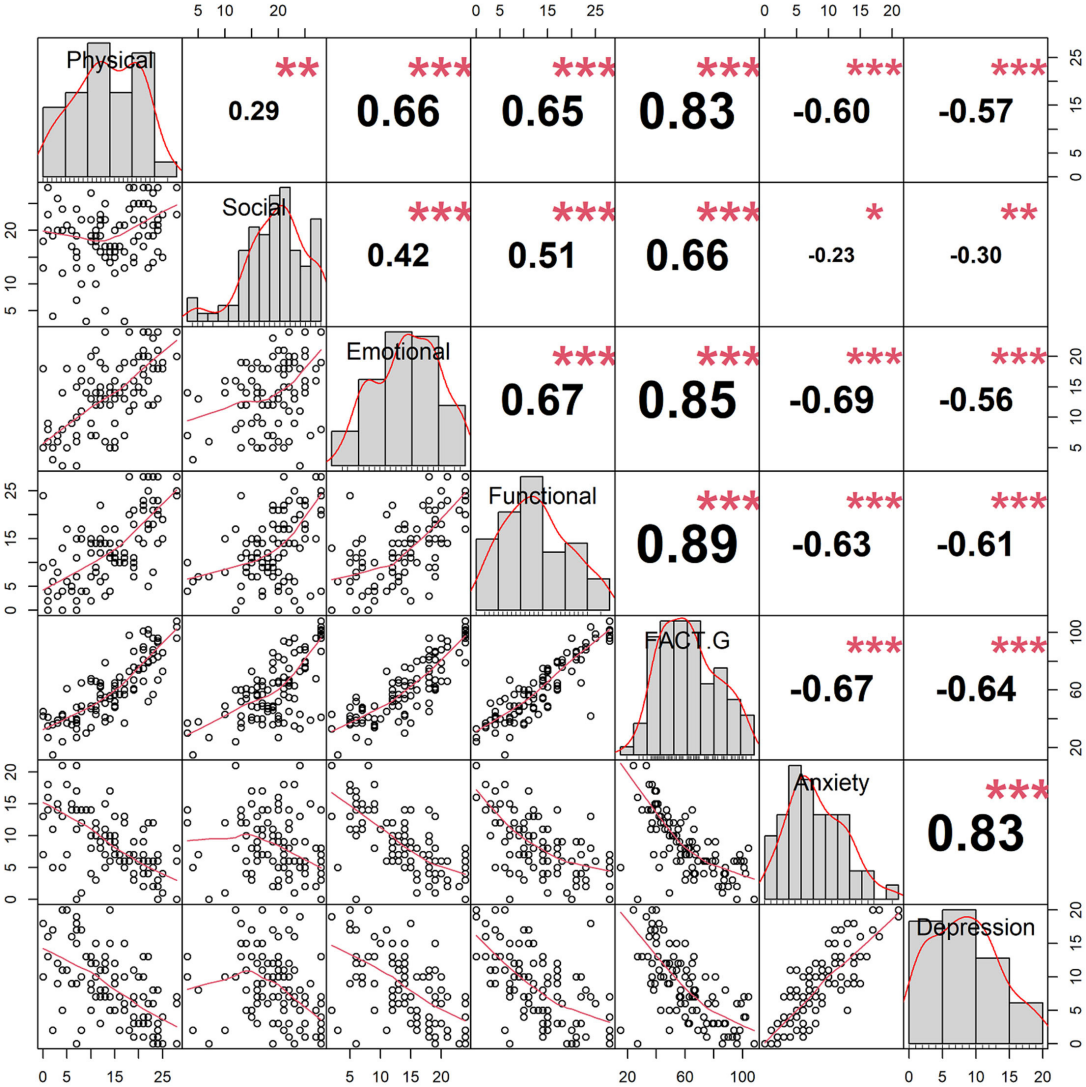
Correlation matrix for scales, including the FACT-G scale and its subscales, the anxiety scale, and the depression scale. Lower left panels show the scatted diagrams between scales and smooth lines (red) were fitted. Upper right panels show the correlation coefficients between scales (* indicates *p* < 0.05, ** indicates *p* < 0.01, *** indicates *p* < 0.001). Diagonal panels show histograms of each scale.

Figure 5 shows the correlation between physical wellbeing, social wellbeing, emotional wellbeing, functional wellbeing, FACT-G, anxiety, and depression scores. It demonstrated that all scores were significantly correlated. FACT-G and its subscales were negatively correlated with anxiety and depression scores. Anxiety and depression scores were positively correlated. More details about the scatter diagrams and correlation coefficients between those scales are also shown in Figure 5. The multiply linear regression models were also used to assess the correlations of each score after adjusting for patient's age, sex, number of comorbidities, and primary cancer type. After adjustment of the four characteristics, the results also showed that the FACT-G, HADS, and their subscales were significantly and mutually correlated (*p* ≦ 0.02, Table 4).

**Table 4:** Correlations (estimates) between quality of life, anxiety, and depression after adjusting for patient's age, sex, number of comorbidities, and primary cancer type.

**Scores**	**Physical wellbeing**	**Social/family wellbeing**	**Emotional wellbeing**	**Functional wellbeing**	**Total FACT-G score**	**HADS-anxiety**	**HADS-depression**
Physical wellbeing	1.00	0.35^*^	0.78^*^	0.62^*^	0.28^*^	−0.93^*^	−0.77^*^
Social/family wellbeing	0.35^*^	1.00	0.40^*^	0.39^*^	0.18^*^	−0.29^**^	−0.33^*^
Emotional wellbeing	0.78^*^	0.40^*^	1.00	0.53^*^	0.23^*^	−0.88^*^	−0.63^*^
Functional wellbeing	0.62^*^	0.39^*^	0.53^*^	1.00	0.31^*^	−0.98^*^	−0.85^*^
Total FACT-G score	0.28^*^	0.18^*^	0.23^*^	0.31^*^	1.00	−3.04^*^	−2.59^*^
HADS-anxiety	−0.93^*^	−0.29^**^	−0.88^*^	−0.98^*^	−3.04^*^	1.00	0.74^*^
HADS-depression	−0.77^*^	−0.33^*^	−0.63^*^	−0.85^*^	−2.59^*^	0.74^*^	1.00

* * Indicates P < 0.01*;

** *indicates P = 0.02. Estimates and P values were derived from the linear regression models. Subtraction sign in the table indicated negative correlation. FACT-G, Functional Assessment of Cancer Therapy-General Scale; HADS, Hospital Anxiety and Depression Scale*.

### Multivariate Analysis of Potential Risk Factors

**Figure 6:**
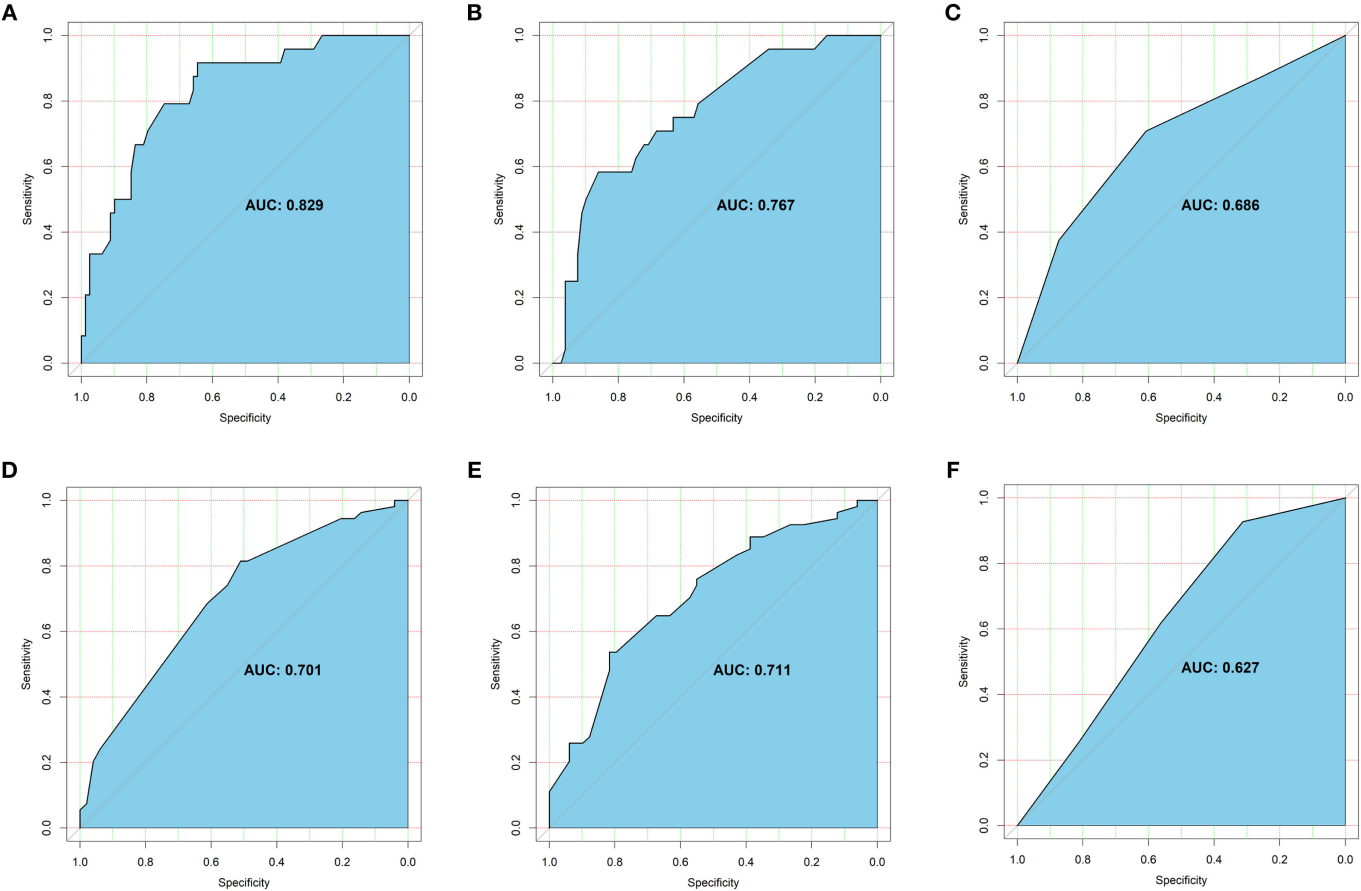
Receiver operating characteristic (ROC) curve for significant variables: **(A)** ROC curve for quality of life when significant variables were combined, including age, preference to eating vegetables, time since knowing cancer diagnosis, surgical treatment at primary cancer, hormone endocrine therapy, and economic burden due to cancer treatments. **(B)** ROC curve for quality of life when age was excluded in the analysis. **(C)** ROC curve for quality of life when age was included in the analysis alone. **(D)** ROC curve for anxiety status when significant variables were combined, including number of comorbidities and economic burden due to cancer treatments. **(E)** ROC curve for anxiety status when age was included in the analysis. **(F)** ROC curve for depression status when age was included in the analysis alone. The sky-blue area was the AUROC and the optimal cutoff was given in each curve.

In the multiple logistic regression models, age (OR = 0.57, 95% confident interval [CI]: 0.38–0.86, *p* < 0.01), preference to eating vegetables (OR = 4.49, 95%CI: 1.21–16.62, *p* = 0.02), time since knowing cancer diagnosis (OR = 1.49, 95%CI: 1.02–2.18, *p* = 0.04), surgical treatment at primary cancer (OR = 0.56, 95%CI: 0.32–0.97, *p* = 0.04), hormone endocrine therapy (OR = 0.27, 95%CI:0.09–0.84, *p* = 0.02), and economic burden due to cancer treatments (OR = 0.40, 95%CI:0.23–0.71, *p* < 0.01) were found to be significantly associated with quality of life (Table 5). The AUROC value of age alone was 0.69, preference to eating vegetables alone was 0.58, time since knowing cancer diagnosis alone was 0.55, surgical treatment at primary cancer alone was 0.58, hormone endocrine therapy alone was 0.51, and economic burden due to cancer treatments alone was 0.62. When the six significant variables combined, the AUROC value was up to 0.83 (Figure 6A). When age was excluded in the analysis, the AUROC decreased to 0.77 (Figure 6B), and when age was included in the analysis alone, the AUROC was 0.69 (Figure 6C).

**Table 5:** Multivariate analyses of the ability of potential risk factors for predicting quality of life, anxiety, and depression among patients with spine metastasis.

**Characteristics**	Quality of life^*^		Anxiety^**^		Depression^**^	
	**OR (95%CI)**	***P***	**OR (95%CI)**	***P***	**OR (95%CI)**	***P***
Age	0.57 (0.38–0.86)	<0.01	1.52 (0.94–2.43)	0.08	1.55 (1.00–2.42)	0.05
Sex	0.86 (0.37–2.00)	0.72	0.81 (0.30–2.15)	0.67	0.82 (0.32–2.11)	0.67
Education level	1.08 (0.67–1.72)	0.76	1.09 (0.62–1.89)	0.77	0.96 (0.56–1.63)	0.87
Care giver	0.76 (0.51–1.14)	0.19	1.41 (0.85–2.32)	0.18	1.48 (0.93–2.36)	0.10
Preference to eating meat	1.74 (0.72–4.21)	0.22	0.75 (0.26–2.15)	0.59	1.25 (0.45–3.48)	0.68
Preference to eating vegetables	4.49 (1.21–16.62)	0.02	2.06 (0.43–9.94)	0.37	1.04 (0.25–4.31)	0.96
Preference to eating fruits	1.43 (0.63–3.24)	0.39	0.54 (0.20–1.48)	0.23	0.41 (0.16–1.08)	0.07
Addiction to smoking	1.03 (0.57–1.86)	0.92	0.75 (0.37–1.50)	0.41	1.06 (0.54–2.07)	0.86
Addiction to drinking	0.86 (0.39–1.91)	0.71	0.82 (0.32–2.11)	0.68	0.68 (0.27–1.71)	0.41
Number of comorbidities	0.71 (0.45–1.10)	0.13	1.85 (1.05-3.26)	0.03	1.66 (0.98–2.80)	0.06
Time since knowing cancer diagnosis (months)	1.49 (1.02–2.18)	0.04	0.92 (0.59–1.43)	0.70	1.13 (0.73–1.73)	0.59
Primary cancer type	0.96 (0.84–1.10)	0.57	1.06 (0.90–1.25)	0.47	1.04 (0.89–1.21)	0.62
Visceral metastasis	0.55 (0.23–1.35)	0.19	2.34 (0.84–6.55)	0.10	2.68 (0.96–7.50)	0.06
Surgical treatment at primary cancer	0.56 (0.32–0.97)	0.04	1.66 (0.86–3.22)	0.13	1.51 (0.80–2.85)	0.20
Surgical treatment at spine metastasis	1.81 (0.86–3.84)	0.12	0.87 (0.37–2.08)	0.76	0.77 (0.33–1.80)	0.55
Radiotherapy	0.63 (0.24–1.65)	0.35	1.18 (0.38–3.67)	0.78	0.68 (0.23–2.01)	0.49
Chemotherapy	0.53 (0.21–1.33)	0.18	1.89 (0.64–5.63)	0.25	1.69 (0.59–4.83)	0.33
Hormone endocrine therapy	0.27 (0.09–0.84)	0.02	0.50 (0.13–1.98)	0.33	1.39 (0.40–4.88)	0.61
Disposable monthly income	0.65 (0.39–1.08)	0.10	1.33 (0.72–2.45)	0.36	0.67 (0.37–1.20)	0.18
Economic burden due to cancer treatments	0.40 (0.23–0.71)	<0.01	4.32 (2.00–9.32)	<0.01	1.52 (0.79–2.94)	0.21

* * Indicates quality of life was assessed by the FACT-G, which was divided into eight groups in the multiply logistic regression models*;

** *indicates anxiety and depression were assessed by the HADS and both were divided into three groups in the multiply logistic regression models. OR, odds rate; CI, confident interval*.

A number of comorbidities (OR = 1.85, 95%CI: 1.05–3.26, *p* = 0.03) and economic burden due to cancer treatments (OR = 4.32, 95%CI: 2.00–9.32, *p* < 0.01) were significantly relevant to anxiety, and age (OR = 1.52, 95%CI: 0.94–2.43, *p* = 0.08) was also close to significance. The AUROC of number of comorbidities alone was 0.57 and economic burden due to cancer treatments alone was 0.66. When the two significant variables combined, the AUROC was 0.70 (Figure 6D). When age was included in the analysis, the AUROC could increase to 0.71 (Figure 6E).

Only age (OR = 1.55, 95%CI: 1.00–2.42, P = 0.05) was shown to be significantly associated with depression, and preference to eating fruits (OR = 0.41, 95%CI: 0.16–1.08, *p* = 0.07), number of comorbidities (OR = 1.66, 95%CI: 0.98–2.80, *p* = 0.06), and visceral metastasis (OR = 2.68, 95%CI: 0.96–7.50, *p* = 0.06) were very closely related to depression but these variables did not reach significance. The AUROC of age alone was 0.63 (Figure 6F).

## Discussion

This study found that patients with spine metastasis suffered from poor quality of life and severe anxiety and depression. In detail, the mean total FACT-G score was only 61.38 ± 21.26, 52.43% patients had skeptical or identified anxiety, and 53.40% patients suffered from skeptical or identified depression. More explicitly, 33.01% patients had identified anxiety and the same number of patients had identified depression, which were consistent with previous studies (3, 4). Other researchers reported that 23.4–32.0% patients with cancer had anxiety and 19.1–47.0% lived with depression (3, 4).

Whether and how age plays a role in influencing quality of life remains unclear. Nipp et al. (17) found that patients aged <65 years experienced the improved quality of life after early palliative care. Subramaniam et al. (27) found that increasing age was also associated with poor health-related quality of life after analyzing 1,490 newly-diagnosed patients with cancer and following up for 1 year. Pan et al. (28) found that diagnosis at an older age was significantly associated with poor quality of life among children and adolescents with cancer. However, several studies also showed that age had no significant effects on affecting patient's quality of life and mental issues (16, 20, 29). Even a study conducted by Zhang et al. (30) found that increasing age was significantly associated with better quality of life among patients with breast cancer-related lymphedema. This difference might be capable of being explained by population heterogeneities, inconsistent evaluation tools, and sample sizes. Notably, this difference might be attributable to the fluctuations of quality of life with changes of age. This study was the first to point out that patients had an age of 60 or more and <70 years had the poorest quality of life and the most serious anxiety and depression status. To begin with, the quality of life was declined with the increasing age, reached its bottom at the age of 60 or more and <70 years, and then increased with the growth of age. Thus, early mental health interventions and effective cares, such as appropriate physical activities, health lifestyles, and proper management of moods, should be conducted to the advanced cancer patients with spine metastases, especially among those with an age of 60 or more and <70 years. Treating with surgery, if applicable, may be able to boost quality of patient's remaining life (10, 11).

The Cronbach's α coefficient of the FACT-G, HADS, and their subscales was above 0.8, which indicated that the internal consistency was good in our Chinese population. After adjustment, the FACT-G, HADS, and their subscales were also significantly and mutually correlated. This is reasonable that patients who suffer from severer mental disorders are more likely to have a poorer quality of life. A study also demonstrated that anxiety and depression negatively correlated with patient's quality of life (31). Similarly, our study also found that the FACT-G and its four subscales were negatively associated with HADS anxiety and HADS depression. More explicitly, HADS scores increased with patient's age, reached its spike, and then decreased as age growing, which was the opposite trend of the scores of the quality of life. However, several studies showed that the younger tended to be more anxious in patients with cancer (32) and higher emotional stress in some types of cancer after diagnosis of cancer (15). This needs to be further investigated in the future.

When adjusted for other potential risk factors, age was also shown to be significantly with quality of life and depression. Besides, we also found that preference to eating vegetables, time since knowing cancer diagnosis, surgical treatment at primary cancer, hormone endocrine therapy, and economic burden due to cancer treatments were found to be significantly associated with quality of life. According to the results, this was to say that good life style (preference to eating vegetables), longer time since knowing cancer diagnosis, surgical treatment at primary cancer site, no need to receive hormone endocrine therapy, and declining economic burden due to cancer treatments could contribute to better quality of life. When patients initially knew the fact of his or her cancer diagnosis, the patients would seriously suffer from negative behavior and mental changes and then gradually tended to accept and adopt to the conditions, which might explain longer time since knowing cancer diagnosis would relate to better patient's quality of life. Surgical treatment at primary cancer site usually meant that the patients had relatively good prognosis and life expectancy. By contrast, those who did not receive surgery at primary cancer site usually failed to have a chance or opportunity to receive surgery probably due to extensive cancer metastases. Fontes et al. (33) pointed out that the breast cancer patient's quality of life was remarkably improved after surgery. Bendixen et al. (34) also found that video-assisted thoracoscopic surgery for lung cancer boosted the improvement of patient's quality of life. Our study also showed that not receiving hormone endocrine therapy was relevant to better quality of life and this might be explained by the side effects of hormone endocrine therapy, negatively affecting patient's quality of life. Severe economic burden due to cancer treatments was significantly associated with poor quality of life and serious anxiety. Of note, more than half of patients reported that cancer treatments triggered severe economic pressure. For one thing, our survey was performed during the great COVID-19 pandemic which contributed to profound the impacts on patient's life due to lockdown and self-isolation. It was reported that the great pandemic had already led to emotional distress, unaffordability, and declining quality of life among patients with cancer (35). For another thing, high medical costs from new adjuvant therapy for patients with cancer, such as immune therapies and bio-target therapies, could lead to heavy economic burdens on these patients and their family members. Mehlis et al. (36) also showed that high financial loss was significantly with poor patient's quality of life and more distress, which was consistent with our present study. The identification of new modifiable risk factors for poor outcome especially among patients with metastatic spinal disease, such as eating habits, surgical treatments, and economic issues, could help doctors and patients to make better clinical decisions to improve patient's quality of life and mental health status. Furthermore, several studies reported that art therapy (37), aerobic exercise (38), and cognitive behavioral therapy (39) might also can play an important role in alleviating cancer patient's anxiety and depression and improving their quality of remaining life.

## Limitations

First, this study could not identify causal relationship because it was a cross-sectional study in nature, but this study was the first to point out the detailed relationship between quality of life and age among advanced patients with spine metastases. Second, the sample size of the study was not large enough which might trigger biases, but, to the best of our knowledge, this study had the largest sample in the current literature about the quality of life and mental health, especially among advanced cancer patients with spine metastases. Besides, carefully and comprehensive statistical analysis was conducted in the study. Several new risk factors associating with quality of life and mental health status especially among spine metastasis patients were identified in the study. Third, this study only evaluated the quality of life and mental health status once, thus, the dynamic changes in quality of life and mental health still remained unclear. In the future, patient's routine follow-ups would give a clear vision about the dynamic changes in these outcomes among cancer patients with metastatic spinal disease.

## Conclusions

Advanced cancer patients with spine metastases suffer from poor quality of life and severe anxiety and depression, especially among patients with an age of 60 or more and less than 70 years. Early mental health care and effective measures should be conducted to advanced cancer patients with spine metastases, and more attention should be paid to take care of patients with an age of 60 or more and <70 years in terms of their quality of life and mental health status.

## Data Availability Statement

The raw data supporting the conclusions of this article will be made available by the authors, without undue reservation.

## Ethics Statement

The studies involving human participants were reviewed and approved by the Ethics Committee of the Fourth Medical Center of Chinese PLA General Hospital. The patients/participants provided their written informed consent to participate in this study.

## Author Contributions

YL, XC, and ML conceived and designed this study together. ML and XZ undertook the data analysis, result interpretation, and manuscript preparation. YL, XS, and HQ performed supervision. All authors read and approved the final manuscript.

## Conflict of Interest

The authors declare that the research was conducted in the absence of any commercial or financial relationships that could be construed as a potential conflict of interest.

## Publisher's Note

All claims expressed in this article are solely those of the authors and do not necessarily represent those of their affiliated organizations, or those of the publisher, the editors and the reviewers. Any product that may be evaluated in this article, or claim that may be made by its manufacturer, is not guaranteed or endorsed by the publisher.

## Supplementary Material

Supplementary Figure S1 Patient's flowchart.

Supplementary Figure S2 The relationship between age and quality of life (FACT-G scores). Age was plotted against FACT-G scores with scatter points and a fitted linear (FACT-G scores = −0.4047^*^Age + 85.35). Red line indicates the fitted linear. Gray indicates the 95% confident interval of the fitted linear.
