# Supplementary figures and images for: Quality of Life and Mental Health Status Among Cancer Patients With Metastatic Spinal Disease
Source: Front Public Health. 2022 Jul 5;10:916004. doi: 10.3389/fpubh.2022.916004 (PMC9294283; doi:10.3389/fpubh.2022.916004)

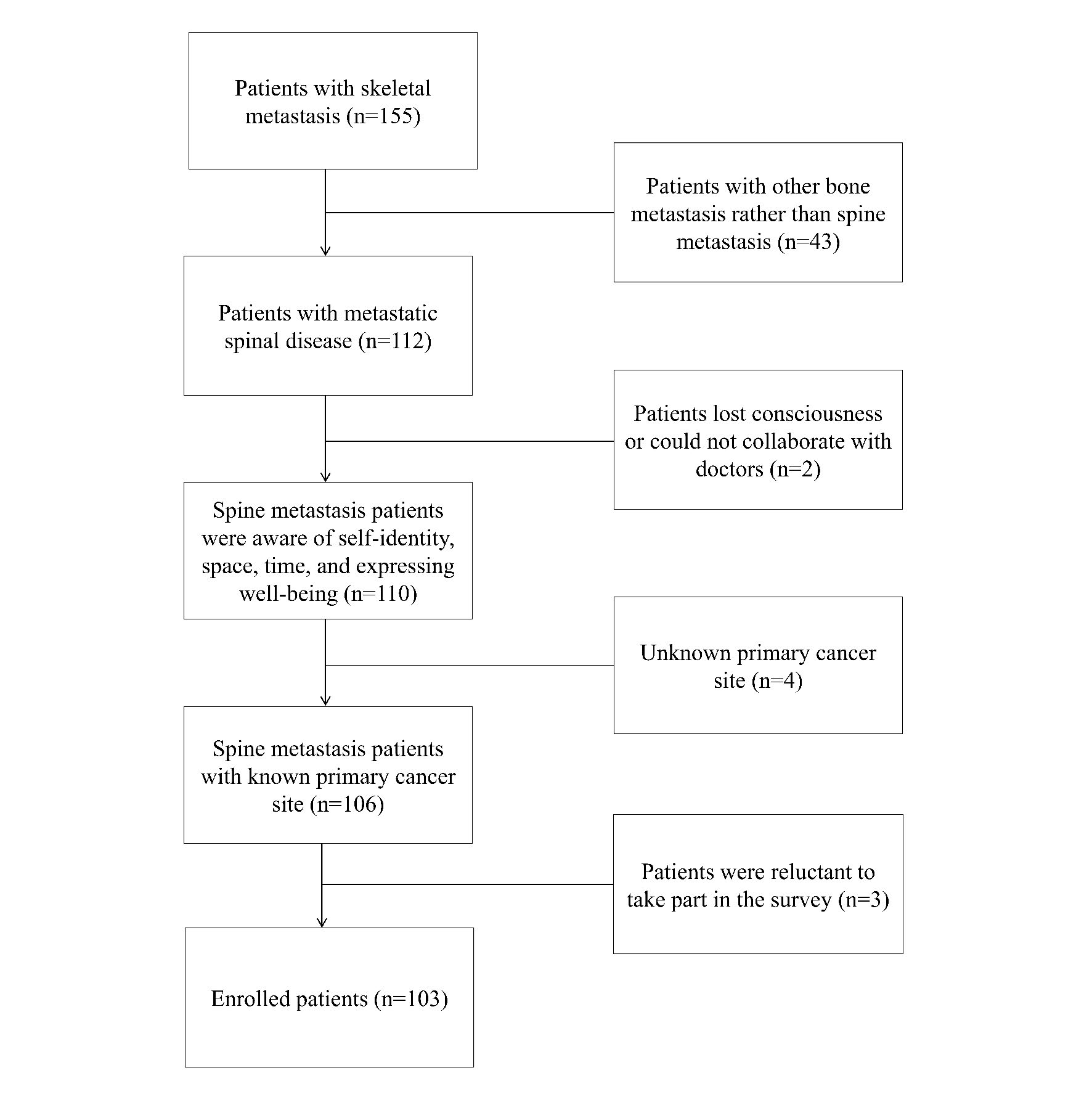

Supplement: Supplementary Figure S1 — Patient's flowchart. [file Image_1.TIF]

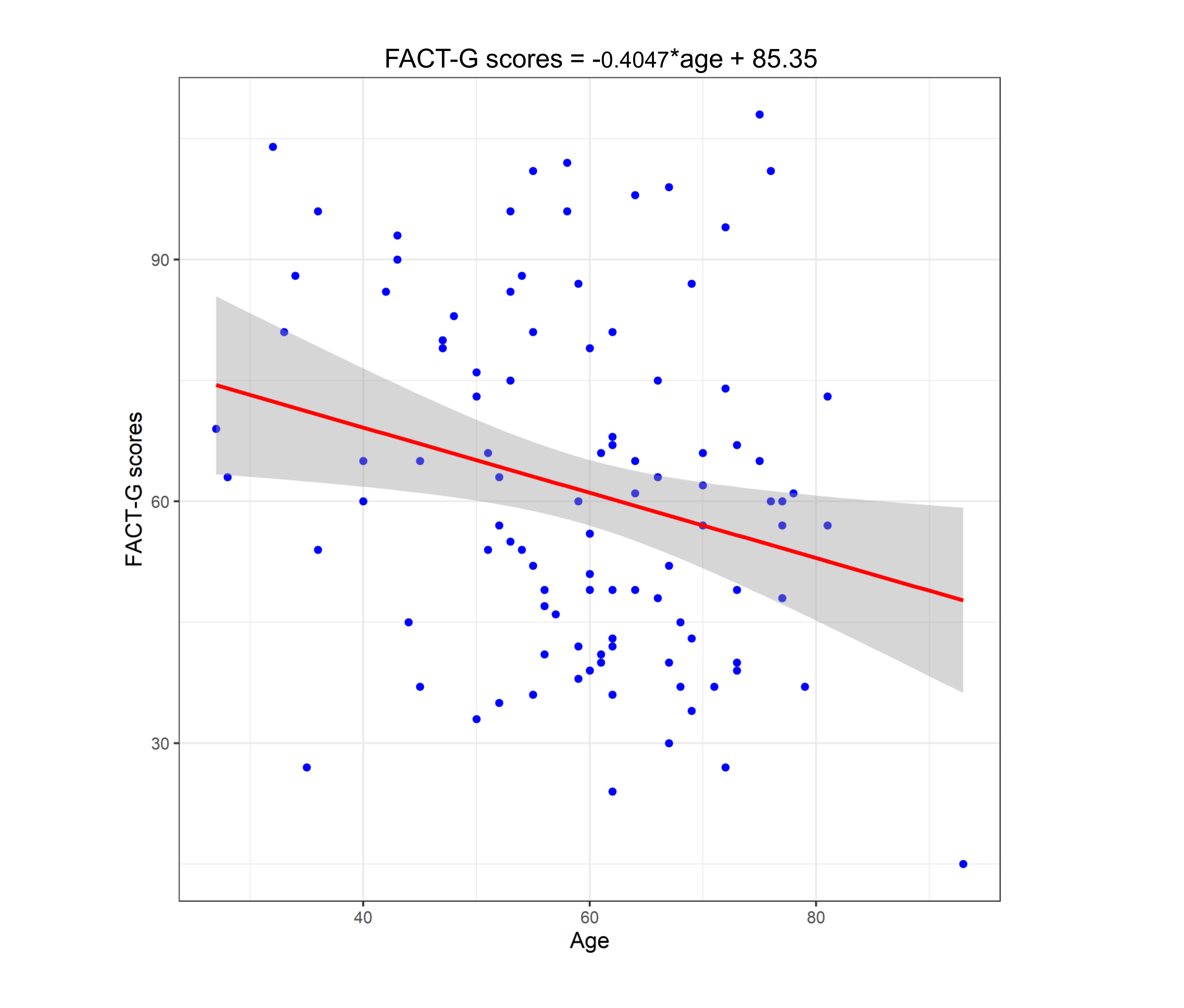

Supplement: Supplementary Figure S2 — The relationship between age and quality of life (FACT-G scores). Age was plotted against FACT-G scores with scatter points and a fitted linear (FACT-G scores = −0.4047*Age + 85.35). Red line indicates the fitted linear. Gray indicates the 95% confident interval of the fitted linear. [file Image_2.TIF]
