# Supplementary material for: Quality of Life and Mental Health Status Among Cancer Patients With Metastatic Spinal Disease
Source: Front Public Health. 2022 Jul 5;10:916004. doi: 10.3389/fpubh.2022.916004 (PMC9294283; doi:10.3389/fpubh.2022.916004)
Supplement: Supplementary file 3 [file Data_Sheet_1.PDF]

Quality of Life and Mental Health Status among Cancer Patients with Metastatic Spinal Disease

Dear Participants,

We are sincerely inviting you to participate in this survey entitled "*Quality of Life and Mental Health Status among Cancer Patients with Metastatic Spinal Disease*". This survey aims to investigate the quality of life and mental health status among advanced cancer patients with metastatic spinal disease and further to identify relevant risk factors. Under the guide of related predictors, appropriate healthcare interventions can be individually conducted. A comprehensive knowledge on quality of life and mental health status would be of great help to information sharing between clinicians and patients and their family members.

The questionnaire is comprised of 20 questions and two scales and it takes about 15 minutes to complete. You are asked to be totally voluntary to participate in the survey and you have rights to refuse the survey at any time under any reasons. Please response all the questions according to your actual conditions. The survey can only be submitted if you complete and answer all the questions entirely.

Thank you for your assistance in this important endeavor.

Best wishes,

Yaosheng Liu on behalf of all investigators.

Senior Department of Orthopedics, the Fourth Medical Center of Chinese PLA General Hospital. No.8, Fengtaidongda Rd, Beijing 100071, People's Republic of China. E-mail address: 15810069346@qq.com. Telephone number: 86-15810069346.

Statement

☐ I have read and understood the informed consent and volunteer to participate in the survey.

☐ I decline to participate in this study.

Questions

1. You are _____ years old.

---

2. What is your gender?

☐ Male
☐ Female

---

3. What is your education level?

☐ Primary or junior school
☐ High school
☐ Undergraduate
☐ Graduate student or above

---

4. Who is your caregiver?

☐ Spouse
☐ Other family member
☐ Nursing worker
☐ None

---

5. Do you prefer to eating meat?

☐ Yes
☐ No

---

6. Do you prefer to eating vegetables?

☐ Yes
☐ No

---

7. Do you prefer to eating vegetables?

☐ Yes
☐ No

---

8. Do you addict to smoking?

☐ Yes
☐ Abstain from smoking
☐ No

---

9. Do you addict to drinking?

☐ Yes

☐ Abstain from drinking
☐ No

---

10. Have you ever diagnosed with the following comorbidities? (Multiple-choice question)

☐ None
☐ Hypertension
☐ Diabetes
☐ Coronary heart disease
☐ Cataract
☐ Glaucoma
☐ Chronic liver disease
☐ Benign prostatic hyperplasia
☐ Chronic kidney disease
☐ Cerebral vascular disease
☐ Asthma
☐ Chronic bronchitis
☐ Others

---

11. Time since knowing cancer diagnosis (months)

☐ ＜3
☐ ≧3 and ＜6
☐ ≧6 and ＜12
☐ ≧12

---

12. What is your primary cancer type?

☐ Lung cancer
☐ Liver cancer
☐ Breast cancer
☐ Prostate cancer
☐ Digestive tract cancer
☐ Others

---

13. Do you have visceral metastasis?

☐ Yes
☐ No

---

14. Do you have surgical treatment at primary cancer?

☐ Open surgery
☐ Minimally invasive surgery
☐ None

---

15. Do you have surgical treatment at spine metastasis?

☐ Open surgery

☐ Minimally invasive surgery
☐ None

16. Have you ever received radiotherapy?

☐ Yes
☐ No

17. Have you ever received chemotherapy?

☐ Yes
☐ No

18. Have you ever received hormone endocrine therapy?

☐ Yes
☐ No

19. What is your disposable monthly income (Yuan, Renminbi (RMB))?

☐ ＜5000
☐ ≧5000 and ＜10000
☐ ≧10000 and ＜20000
☐ ≧20000

20. What is your economic burden due to cancer treatments?

☐ None
☐ Mild
☐ Moderate
☐ Severe

The Functional Assessment of Cancer Therapy - General (FACT-G)

Please mark one box per question to indicate your response as it applies to the past 7 days.

	PHYSICAL WELL-BEING	Not at all	A little bit	Some-what	Quite a bit	Very much
**1**	I have a lack of energy	☐	☐	☐	☐	☐
**2**	I have nausea	☐	☐	☐	☐	☐
**3**	Because of my physical condition, I have trouble meeting the needs of my family	☐	☐	☐	☐	☐
**4**	I have pain	☐	☐	☐	☐	☐
**5**	I am bothered by side effects of treatment					

**6**	I feel ill	☐	☐	☐	☐	☐
**7**	I am forced to spend time in bed	☐	☐	☐	☐	☐

	**SOCIAL/FAMILY WELL-BEING**	**Not at all**	**A little bit**	**Some-what**	**Quite a bit**	**Very much**
**1**	I feel close to my friends	☐	☐	☐	☐	☐
**2**	I get emotional support from my family	☐	☐	☐	☐	☐
**3**	I get support from my friends	☐	☐	☐	☐	☐
**4**	My family has accepted my illness	☐	☐	☐	☐	☐
**5**	I am satisfied with family communication about my illness					
**6**	I feel close to my partner (or the person who is my main support)	☐	☐	☐	☐	☐
**7**	I am satisfied with my sex life	☐	☐	☐	☐	☐

	**EMOTIONAL WELL-BEING**	**Not at all**	**A little bit**	**Some-what**	**Quite a bit**	**Very much**
**1**	I feel sad	☐	☐	☐	☐	☐
**2**	I am satisfied with how I am coping with my illness	☐	☐	☐	☐	☐
**3**	I am losing hope in the fight against my illness	☐	☐	☐	☐	☐
**4**	I feel nervous	☐	☐	☐	☐	☐
**5**	I worry about dying					
**6**	I worry that my condition will get worse	☐	☐	☐	☐	☐

	**FUNCTIONAL WELL-BEING**	**Not at all**	**A little bit**	**Some-what**	**Quite a bit**	**Very much**
**1**	I am able to work (include work at home)	☐	☐	☐	☐	☐
**2**	My work (include work at home) is fulfilling	☐	☐	☐	☐	☐
**3**	I am able to enjoy life	☐	☐	☐	☐	☐
**4**	I have accepted my illness	☐	☐	☐	☐	☐
**5**	I am sleeping well					
**6**	I am enjoying the things I usually do for fun	☐	☐	☐	☐	☐
**7**	I am content with the quality of my life right now	☐	☐	☐	☐	☐

Hospital Anxiety and Depression Scale (HADS)

Please mark the box beside the reply that is closest to how you have been feeling in the past 7 days. Don’t take too long over you replies: your immediate is best.

**I feel tense or ‘wound up’:**		**I feel as if I am slowed down:**	
☐	3 Most of the time	☐	3 Nearly all the time
☐	2 A lot of the time	☐	2 Very often
☐	1 From time to time, occasionally	☐	1 Sometimes
☐	0 Not at all	☐	0 Not at all
**I still enjoy the things I used to enjoy:**		**I get a sort of frightened feeling like ‘butterflies’ in the stomach:**	
☐	0 Definitely as much	☐	0 Not at all
☐	1 Not quite so much	☐	1 Occasionally
☐	2 Only a little	☐	2 Quite often
☐	3 Hardly at all	☐	3 Very often
**I get a sort of frightened feeling as if something awful is about to happen:**		**I have lost interest in my appearance:**	
☐	3 Very definitely and quite badly	☐	3 Definitely
☐	2 Yes, but not too badly	☐	2 I don't take as much care as I should
☐	1 A little, but it doesn't worry me	☐	1 I may not take quite as much care
☐	0 Not at all	☐	0 I take just as much care as ever
**I can laugh and see the funny side of things:**		**I feel restless as I have to be on the move:**	
☐	0 As much as I always could	☐	3 Very much indeed
☐	1 Not quite so much now	☐	2 Quite a lot
☐	2 Definitely not so much now	☐	1 Not very much
☐	3 Not at all	☐	0 Not at all
**Worrying thoughts go through my mind:**		**I look forward with enjoyment to things:**	
☐	3 A great deal of the time	☐	0 As much as I ever did
☐	2 A lot of the time	☐	1 Rather less than I used to
☐	1 From time to time, but not too often	☐	2 Definitely less than I used to
☐	0 Only occasionally	☐	3 Hardly at all
**I feel cheerful:**		**I get sudden feelings of panic:**	
☐	3 Not at all	☐	3 Very often indeed
☐	2 Not often	☐	2 Quite often
☐	1 Sometimes	☐	1 Not very often
☐	0 Most of the time	☐	0 Not at all
**I can sit at ease and feel relaxed:**		**I can enjoy a good book or radio or TV program:**	
☐	0 Definitely	☐	0 Often
☐	1 Usually	☐	1 Sometimes
☐	2 Not Often	☐	2 Not often
☐	3 Not at all	☐	3 Very seldom
